# Computational and experimental studies of salvianolic acid A targets 3C protease to inhibit enterovirus 71 infection

**DOI:** 10.3389/fphar.2023.1118584

**Published:** 2023-03-02

**Authors:** Sai Shi, Lei Xie, Sen Ma, Binghong Xu, Hailong An, Sheng Ye, Yaxin Wang

**Affiliations:** ^1^ Tianjin Key Laboratory of Function and Application of Biological Macromolecular Structures, School of Life Sciences, Tianjin University, Tianjin, China; ^2^ Key Laboratory of Molecular Biophysics of Hebei Province, Institute of Biophysics, Hebei University of Technology, Tianjin, China

**Keywords:** HFMD, EV71, antiviral, salvianolic acid A, inhibitor

## Abstract

Hand, foot, and mouth disease (HFMD) is a common childhood infectious disease caused by enterovirus (EV) infection. EV71 is one of the major pathogens causing hand, foot, and mouth disease and is more likely to cause exacerbation and death than other enteroviruses. Although a monovalent vaccine for EV71 has been developed, there are no clinically available anti-EV71 specific drugs. Here, we performed virtual screening and biological experiments based on the traditional Chinese medicine monomer library. We identified a traditional Chinese medicine monomer, Salvianolic acid A (SA), a polyphenolic compound isolated from *Salvia miltiorrhiza*. Salvianolic acid A inhibits EV71 virus infection in a concentration-dependent manner, and its antiviral activity is higher than that of other reported natural polyphenols and has a high biosafety. Furthermore, molecular dynamics simulations showed that salvianolic acid A can anchor to E71, a member of the enzyme catalytic triad, and cause H40 to move away from the catalytic center. Meanwhile, molecular mechanics generalized born surface area (MMGBSA) and steered molecular dynamics (SMD) results showed that the P1 group of SA was most easily unbound to the S1 pocket of 3C^pro^, which provided theoretical support to further improve the affinity of salvianolic acid A with 3C^pro^. These findings suggest that salvianolic acid A is a novel EV71 3C^pro^ inhibitor with excellent antiviral activity and is a promising candidate for clinical studies.

## Graphical Abstract

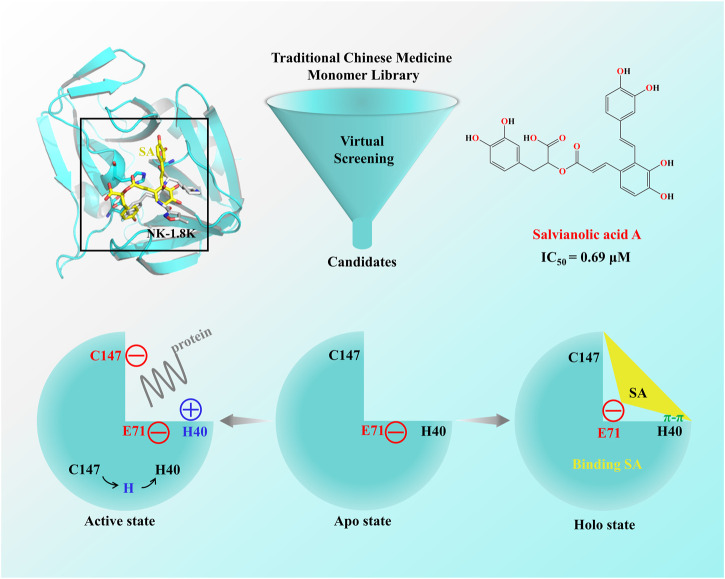

## Introduction

Hand, foot, and mouth disease (HFMD) is an infectious disease caused by enteroviruses that primarily affects infants and young children (Chan et al., 2003). The clinical features are vesicular eruptions mainly on the skin of the hands, feet, and oral cavity, and accompanied by fever (Chen et al., 2007). Enterovirus 71 (EV71) and coxsackievirus A6 (CA6) and A16 (CA16) are the main prevalent pathogens of HFMD. A statistical report from Beijing, China, showed that CA6, CA16, and EV71 were detected in 36.1, 24.1, and 12.0% of the 440 HFMD clusters in 2016–2020, respectively (Cui et al., 2022). EV71 has the highest probability of causing severe illness and death compared to other enteroviruses (Xing et al., 2014). Indeed, EV71 has been associated with a wide spectrum of acute central nervous system (CNS) syndromes, including aseptic meningitis, brain-stem encephalitis, and fulminant neurogenic pulmonary edema (McMinn, 2002). Over the past 20 years, HFMD caused by EV71 has become a major public health challenge throughout the Asia-Pacific region, and the magnitude and severity of the HFMD have caused global concern (Zeng et al., 2012). To prevent a pandemic, China has successfully developed an inactivated monovalent EV71 vaccine (Lin et al., 2019). However, there is still a lack of safe and reliable treatment for patients infected with EV71 (Diarimalala et al., 2020), and the risk of being permanently disabled or fatal after EV71 infection remains, there is a pressing need to develop anti-EV71 drugs to combat HFMD.

EV71 is a non-enveloped virus whose genome is a single positive-stranded RNA that encodes a 5′-UTR, a polyprotein, and a 3′UTR (Solomon et al., 2010). EV71 has been classified into subtypes A, B, C, and D based on the phylogenetics of its major antigenic protein, VP1. Among these, subtype A contains only the prototype strain BrCr, subtypes B and C each have five different subgenogroups (B1–B5 and C1–C5), and the strains circulating in China belong to subtype C4, subtype D is represented by a single strain which has been isolated from India (Lei et al., 2015). The polyprotein of EV71 contains three precursor proteins (P1-P3). P1 is in turn cleaved into four viral capsid proteins (VP1-VP4), P2 and P3 are cleaved into seven non-structural proteins (2A–2C, 3A–3D) involved in protein processing and genome replication (Solomon et al., 2010). The viral 3C protein (3C^pro^) is a cysteine protease containing 183 amino acids and E71, H40, and C147 form a conserved catalytic triad of the protease (Wen et al., 2020). 3C^pro^ is involved in the hydrolysis of all seven non-structural proteins of EV71 as well as two structural proteins (VP1, VP3) (Yuan et al., 2018), and also cleaves host proteins related to the immune response, e.g., 3C^pro^ suppresses RIG-I signaling by disrupting the RIG-I-IPS-1 complex and IRF3 nuclear translocation, affecting the innate immune response (Hornung et al., 2006). The central roles played by EV71 3C^pro^ make it a very promising target for antiviral drug development (Cui et al., 2011).

Structure-based drug design and screening based on 3C^pro^ have identified several active compounds with significant inhibitory effects against EV71 infection (Supplementary Figure S1) (Diarimalala et al., 2020). In 2011, Rupintrivir (AG7088), a 3C^pro^ inhibitor of human rhinovirus, was shown to have strong antiviral activity against EV71 3C^pro^ (Wang et al., 2011). Subsequently, we designed and synthesized NK-1.8k (Wang et al., 2015) and NK-1.9k (Wang et al., 2017a) based on this peptidomimetic compound with better stability and drug properties than rupintrivir, and we resolved the complex structures of NK-1.8K and NK-1.9K with 3C^pro^ and elucidated the interaction modes of small molecules with 3C^pro^ (Wang et al., 2017b). Also, we show that these inhibitors have the highest activity and higher selectivity when the three-residue mimics (AG7088) is shortened to a two-residue peptidyl mimics and the inhibitor P1 group is a δ-lactam and the P1′ group is an aldehyde group or a cyanohydrin group (Supplementary Figure S1). Another peptidomimetic inhibitor reported by our group is (1R, 2S, 2′S, 5S)-9, which is one of the most potent 3C^pro^ inhibitors to date (Zhai et al., 2015). However, the presence of cyanohydrin in the structure gives it unstable and toxic properties. Ma et al. (2016) discovered a novel 3C^pro^ inhibitor, DC07090, which can bind 3C^pro^ and reversibly inhibit their protease activity, showing a high potential for drug generation. In addition, several natural products and derivatives have been shown to have low cytotoxicity and potent antiviral activity, including Luteoloside (Cao et al., 2016), Quercetin (Yao et al., 2018), Chrysin and Diisopropyl Chrysin-7-i1 Phosphate (CPI) (Wang et al., 2014). However, the above active small molecules cannot reach the clinical stage due to their poor oral availability or higher toxicity and easy degradation, thus the discovery of antiviral agents that can enter clinical use is the most urgent task for EV71 drug development (Lu et al., 2011; Diarimalala et al., 2020).

**FIGURE 1:**
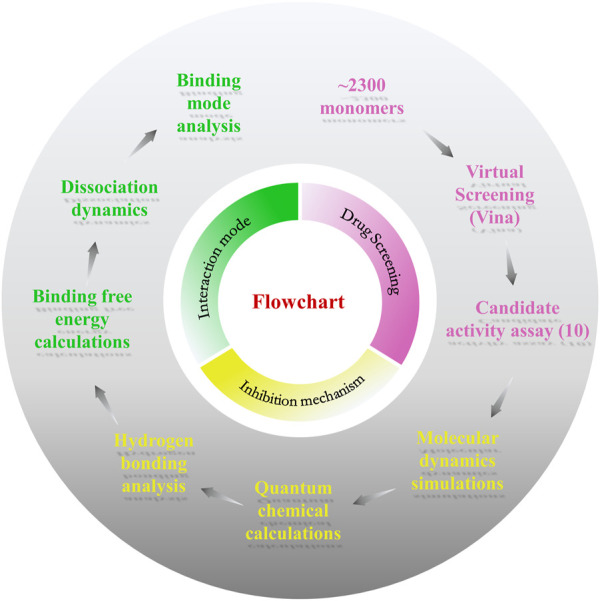
Flowchart of the work. This work consists of three parts: drug screening, inhibition mechanism, and interaction mode.

In this study, we propose a drug screening strategy for traditional Chinese medicine monomer targeting EV71 3C^pro^ (Figure 1). We identified a novel EV71 3C^pro^ inhibitor, Salvianolic acid A (SA) by constructing a traditional Chinese medicine monomer compound library and docking-based virtual screening. We used molecular dynamics simulations and molecular biology experiments to reveal the molecular mechanism of 3C^pro^ inhibition by SA, and tested its antiviral activity by measuring the luciferase expression in cells with EV71 infection. The data show that SA is an EV71 3C^pro^ orthosteric site inhibitor with high antiviral activity. Importantly, two SA-rich Chinese drug agents, DanShenDiWan and FuFangDanShenPian, have been marketed and used for decades as a treatment for angina pectoris, and Danhong injection (containing SA) has entered the clinic for the treatment of stroke in China (Lin et al., 2022), suggesting that SA has good biosafety. Therefore, the development of SA as an antiviral agent would be more economic than innovative drug development. Our strategy offers new ideas for the discovery of safe and effective antiviral drugs.

## Results

### SA is a novel EV71 3C^pro^ inhibitor

**FIGURE 2:**
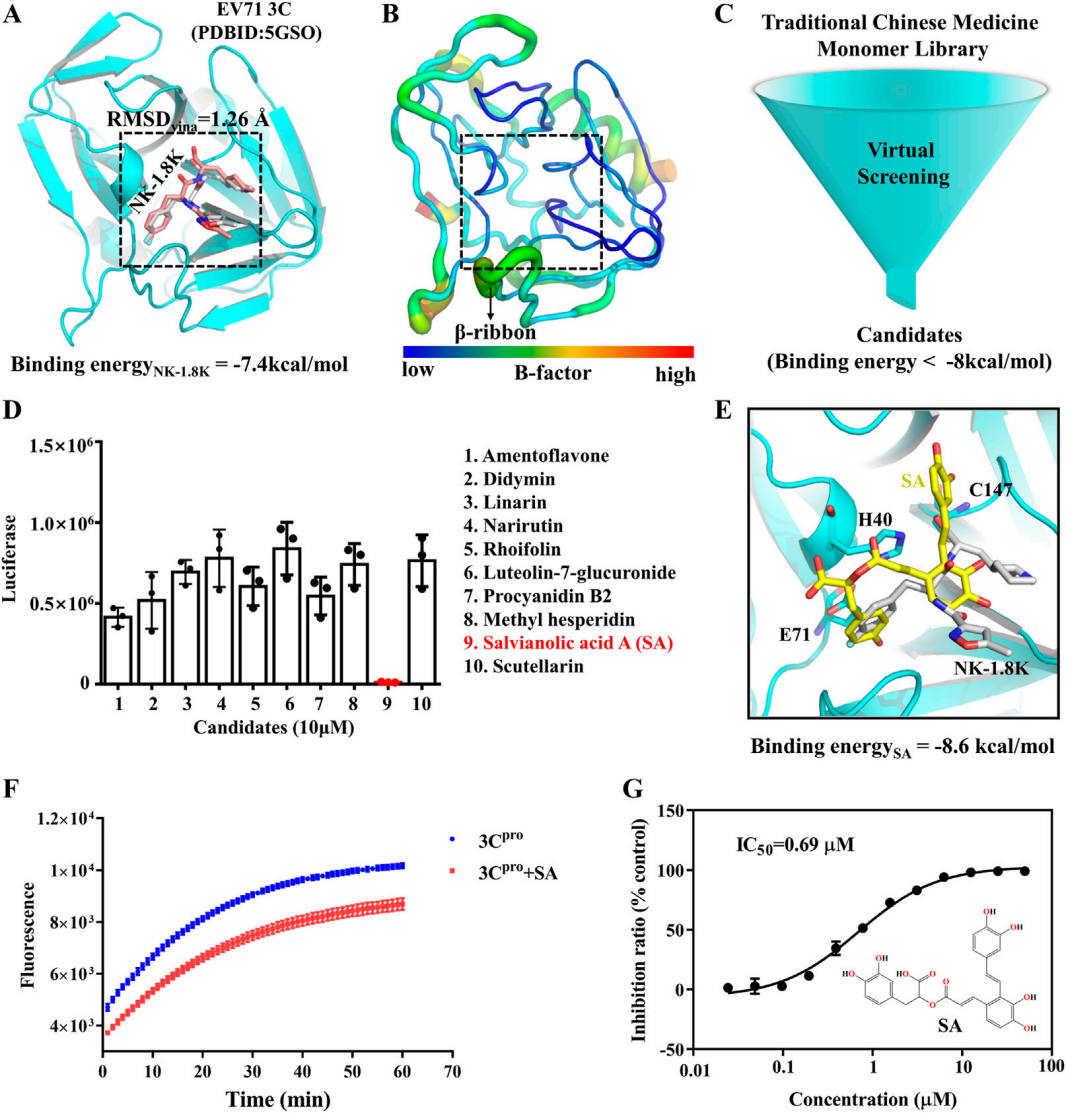
SA is a novel inhibitor of EV71 3C^pro^. **(A)** EV71 3C^pro^ inhibitor binding pocket. **(B)** EV71 3C^pro^ b-factor. **(C)** Virtual Screening flowchart. **(D)** Inhibitory effect of candidates (10 μM) on EV71 infection. **(E)** SA and 3C^pro^ docking conformation. 3C^pro^ is shown in cartoon. SA is shown as yellow sticks. NK-1.8K in the crystal structure is shown as white sticks. **(F)** Fluorescence resonance energy transfer curves of 3C^pro^ hydrolysis substrates. **(G)** IC_50_ of SA inhibition of 3C^pro^ hydrolysis activity. All the data are means ± SD (*n* = 3).

The utilization of traditional Chinese medicines monomers to treat diseases has been a popular research topic for decades and has shown significant curative effects in many cases (Harvey, 2008). To screen antiviral small molecules, we collected more than 2,300 monomers from the Traditional Chinese Medicine Systems Pharmacology Database (Ru et al., 2014). We performed a virtual screen using the previously identified 3C^pro^ inhibitor binding pocket as the receptor docking region. As shown in Figure 2A, the binding pocket was originally an NK-1.8K binding region and was structurally very stable at all sites except for the β-ribbon region (Figure 2B). First, we evaluated the usability of the docking software Vina, and as shown in Figure 2A, the RMSD of the docked conformation of NK-1.8K to the crystal conformation was less than 2 Å, indicating that the scoring function of Vina can accurately describe the ligand binding mode to 3C^pro^. Then, 2,300 monomers compounds were sequentially docked to 3C^pro^. Smaller molecules with higher affinity to proteins (binding energy < −8 kcal/mol) were further screened visually to ensure structural diversity of the molecules (Figure 2C). Finally, 10 candidates were tested for biological activity (Supplementary Table S1). As shown in Figure 2D, 10 μM Salvianolic acid A (SA) almost completely inhibited EV71 infection, indicating its high antiviral activity. The binding energy of SA to 3C^pro^ is −8.6 kcal/mol, and its binding mode is different from that of NK-1.8K (Figure 2E). To verify whether SA targeted at 3C^pro^ and affected its enzymatic activity, we carried out the *in vitro* inhibition assays based on the Fluorescence Resonance Energy Transfer (FRET). The inhibition curves showed that 1 μM of SA significantly reduced the hydrolytic activity of 3C^pro^ (Figure 2F). Then, we quantified the half-inhibitory concentration (IC_50_) of SA inhibition of 3C^pro^, and the IC_50_ value was 0.69 µM (Figure 2G). The activity of SA to inhibit 3C^pro^ is approximately 5.8 times higher than that of chrysin (Wang et al., 2014). Virtual screening and FRET experiments showed that SA is a novel 3C^pro^ inhibitor with higher activity than other natural polyphenols that have been reported (Diarimalala et al., 2020).

**FIGURE 3:**
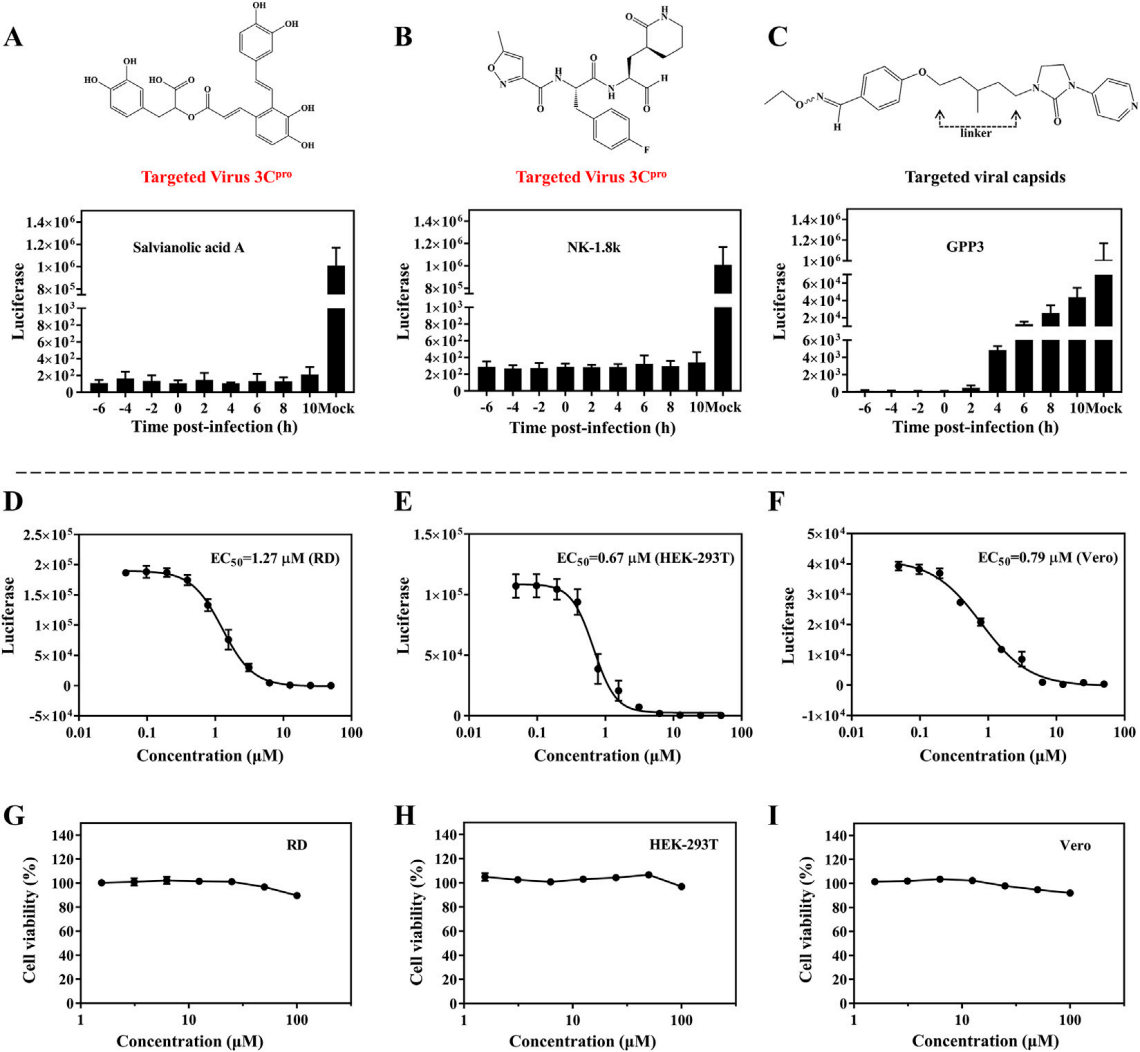
Antiviral activity of SA on different cell lines. **(A–C)** Inhibition of EV71 luciferase reporter virus infection of RD cells by SA (5 μM), NK-1.8k (2 μM), and GPP3 (1 μM) at various addition times (0 hpi indicates the time supplied inhibitors and virus simultaneously). **(D–F)** Quantification of EC_50_ on RD, HEK-293T, and Vero cell lines. **(G–I)** Cytotoxicity of SA on RD, HEK-293T, and Vero cell lines. All the data are means ± SD (*n* = 3).

To explore which infection stages were impacted by SA, we performed time-of-addition assays. The single-round EV71 luciferase virus was used to test virus propagation when treated with SA, which was beneficial to exclude reinfection with the virus. NK-1.8k (targeting EV71 3C^pro^) and GPP3 (targeting the viral capsid) were applied as controls (De Colibus et al., 2015; Wang et al., 2015). NK-1.8k and GPP3 are compounds that inhibit viral replication and entry, respectively. RD cells were infected with EV71 luciferase virus and treated with 5 μM SA, 2 μM NK-1.8k, and 1 μM GPP3 at different time points (−6, −4, −2, 0, 2, 4, 6, 8, and 10 hpi). As shown in Figures 3A, B, the inhibition effect of SA from −6 to 10 hpi were independent of the treatment time. A similar pattern of results was obtained with the viral inhibitor NK-1.8k. Different from the above results, the antiviral effect of the virus entry inhibitor GPP3 was dramatically decreased from 4 hpi (Figure 3C). This experiment showed that SA inhibited virus by the same pattern as NK-1.8K, which cannot inhibit virus entry into cells, but can inhibit virus replication by acting on 3C^pro^. To assess whether the inhibitors’ antiviral activity against this virus depends on the cell types or species, we analyzed the antiviral effect on different cells. The EC_50_ of SA on RD, HEK-293T, and Vero cells were 1.27, 0.67, and 0.79 μM, respectively (Figures 3D–F). This indicates that SA has a significant ability to inhibit EV71 infection on different cell types, and its activity is significantly higher than that of other natural products that have been reported (Diarimalala et al., 2020). Furthermore, we tested the cytotoxicity of SA on RD, HEK-293T, and Vero cells. Even at 100 μM, SA did not affect the viability of the three different cells (Figures 3G–I). Taken together, this evidence suggests that SA has excellent antiviral activity and biological safety *in vitro*.

### The member of the catalytic triad, E71, is the structural basis for SA inhibition of 3C^pro^

**FIGURE 4:**
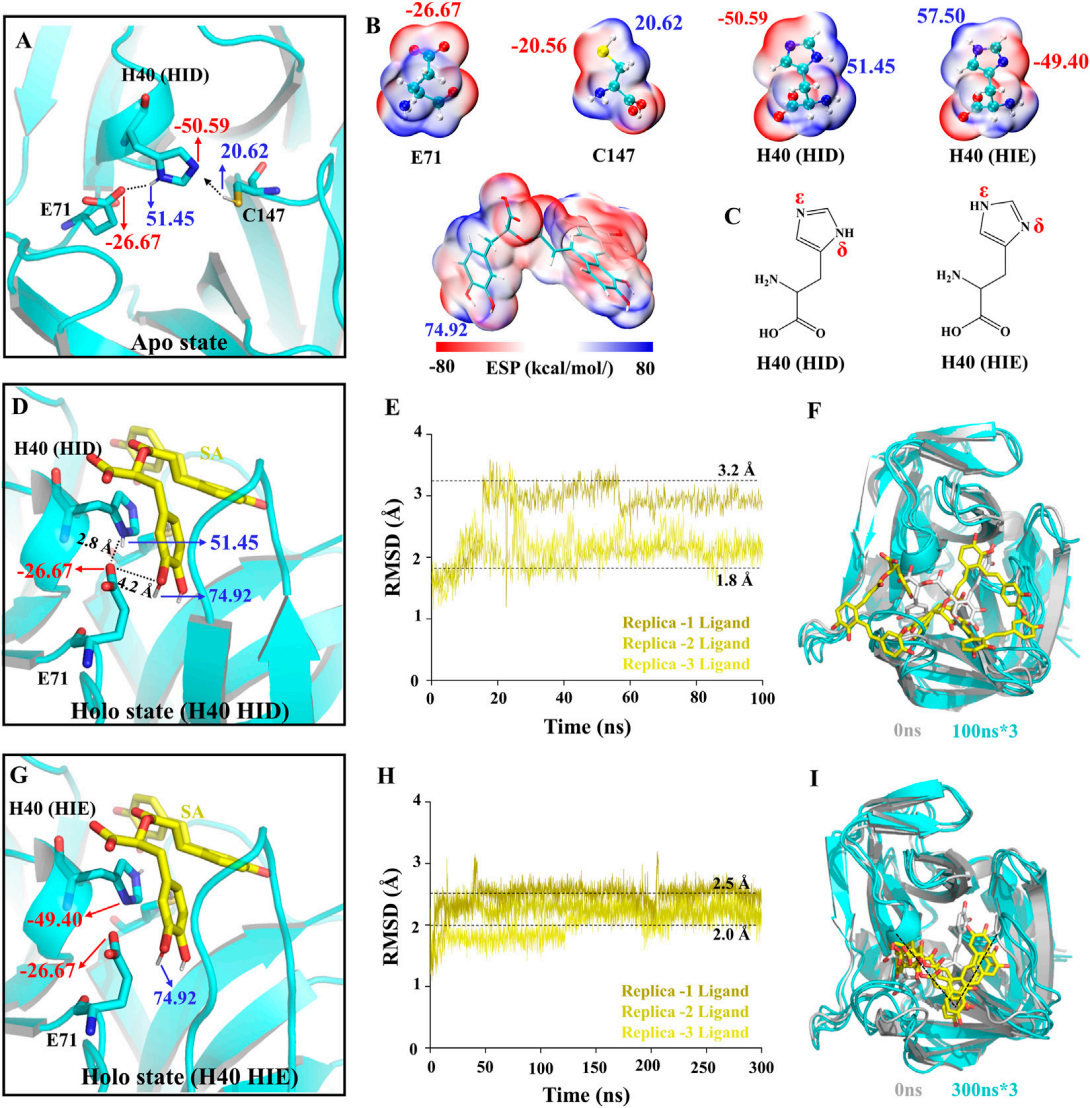
Structural basis of 3C^pro^ activity and construction of simulation system. **(A,D,G)** The charge distribution in the initial state of different simulated systems. **(B)** ESP-mapped molecular VDW surface of E71, H40, C147, and SA. **(C)** Two protonated structural formulae of histidine in the uncharged state. **(E,H)** The root means square deviation (RMSD) of the SA heavy atoms. **(F,I)** The final state of receptors and ligands of the two Holo systems.

The activity of 3C^pro^ depends on the catalytic triad consisting of E71, H40, and C147 (Cui et al., 2011; Wen et al., 2020). First, H40 in the triad exchanges protons with C147, thereby deprotonating C147. Then, the 3C^pro^ reacts with the substrate by acylation to form and release the first product, the amine R-NH_2_. Finally, the acyl-3C^pro^ reacts with a water molecule to release the second product (Yuan et al., 2018). To determine the molecular mechanism of SA inhibition of 3C, we performed conventional molecular dynamics (CMD) simulations (Supplementary Table S2). We calculated the electrostatic surface potential (ESP) of the residues and SA and determined the protonation state of the residues in the simulated system (Figures 4A–C). In the Apo system, E71 forms a hydrogen bond (H-bond) with -NH at the H40 δ site and stabilizes the -N at the H40 ε site pointing to C147. This is the structural basis for the deprotonation of C147 (Figure 4A). However, unlike the Apo system, when we performed protein-ligand complex simulations in the Holo system without any changes, SA was not stabilized in the binding pocket during all three 100 ns simulations and the RMSD of the SA fluctuated drastically (Figures 4D–F). This is understandable because the initial conformation of the protein is from the NK-1.8K complex with 3C^pro^, and the reason for this phenomenon is that the key interaction between the protein and SA is not formed. We calculated the ESP of the key sites in the Holo system and found that SA has two phenolic hydroxyl groups close to the carboxyl group of E71. the ESP of the SA phenolic hydroxyl site is 74.92 kcal/mol, and the ESP of the H40 δ site, which forms a hydrogen bond with E71, is 51.45 kcal/mol (Figure 4G). The binding mode of 3C^pro^ and SA suggests that E71 can only form electrostatic interactions with one of H40 and SA. The ESP values indicate that the potential of the phenolic hydroxyl group of SA is significantly higher than that of the δ-site amino group of H40, so SA may preferentially bind to E71 (Figure 4D). In fact, we also observed the formation of H-bonds between SA and E71 in the Holo simulation system (Supplementary Figure S1). Therefore, for the unaltered Holo system, SA would fall into a meaningless fluctuating state unable to be stabilized. To avoid the system from falling into a meaningless equilibrium, we modified the protonated state of H40 in the Holo system to avoid H40 competing with SA for binding E71 (Figure 4G). We increased the simulation time to 300 ns and analyzed the dynamic behavior of SA in the Holo system. The RMSD of SA and the final conformations of three trajectories showed that the RMSD of SA stabilized in the range of 2–2.5 Å (Figure 4H), and the binding pose was consistent (Figure 4I). This indicated that the interaction mode between SA and 3C^pro^ was more consistent and representative.

**FIGURE 5:**
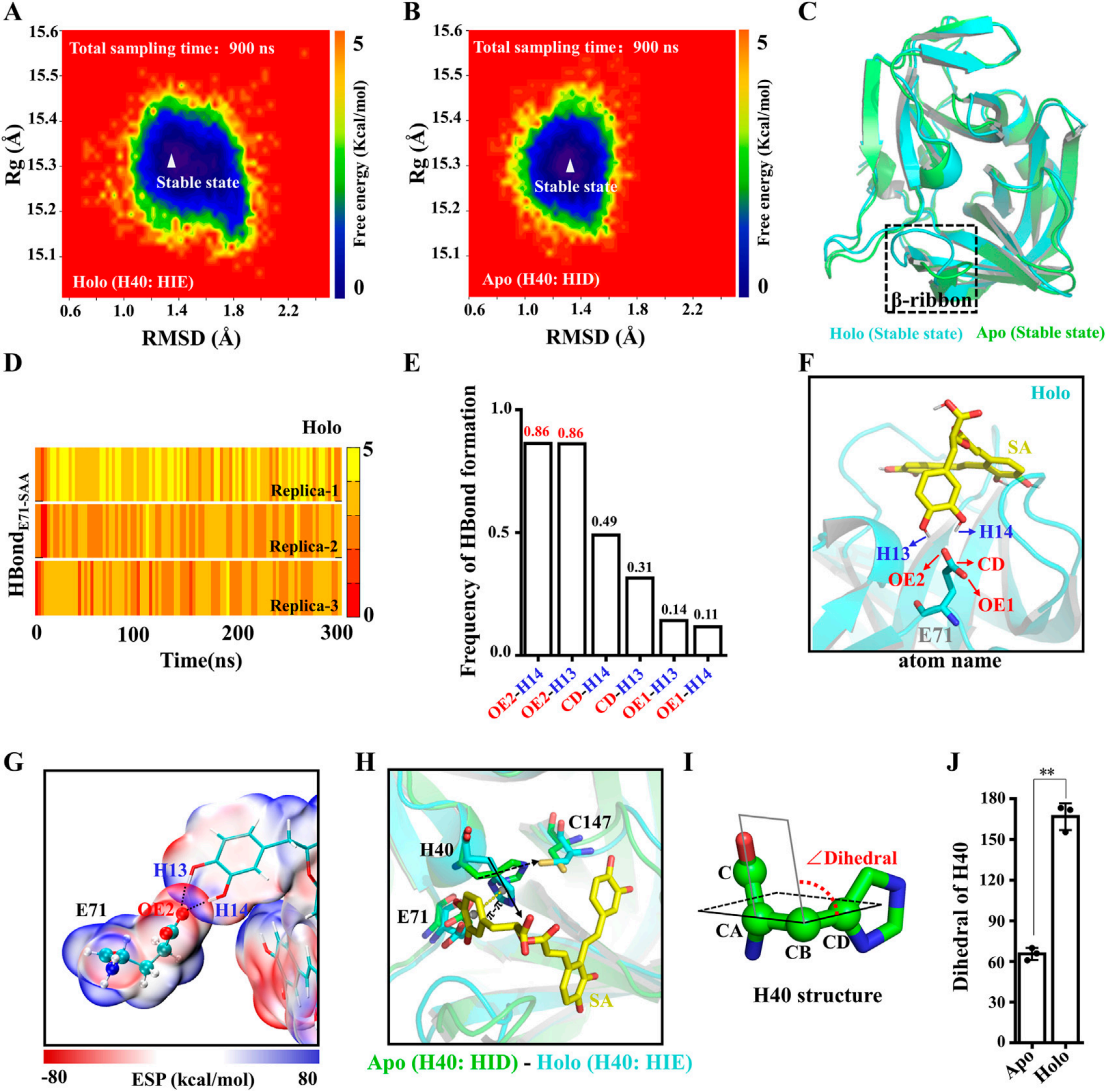
Structural basis of 3C^pro^ inhibition by SA. **(A,B)** Free energy landscape of 3C^pro^ conformations in Apo and Holo systems. **(C)** Overlap of the 3C^pro^ stable state conformations in the Apo and Holo systems. **(D)** Time courses of the H-bond forming between the SA and E71. **(E)** H-bond occupancy between the SA and E71. **(F)** H-bond between SA and E71. **(G)** ESP-mapped molecular VDW surface of SA and E71. The stick represents SA. The sphere model represents residues. **(H)** Representative structure of the final structure of Apo and Holo systems. **(I)** Dihedral angle of H40. **(J)** The absolute value of dihedral of H40 in Apo and Holo systems at 300 ns (*n* = 3; **p < 0.01, Apo vs. Holo).

To evaluate the conformational stability of Apo and Holo systems during MD simulations, we mapped the protein conformational free energy landscape. The RMSD and the radius of gyration (Rg) of the proteins were used as reaction coordinates for the free energy. As shown in Figures 5A, B, the RMSD values of the proteins are distributed in the range of 1.0–1.8 Å and the Rg values are distributed in the range of 15.2–15.4 Å, and there is only one stable state for both systems. Furthermore, we examined the RMSF of the protein in both systems, and the flexibility of the β-ribbon of Apo is significantly higher than that of the Holo system (Supplementary Figures S3A, B), indicating that SA contributes to the stability of this region (Figure 5C). Indeed, the β-ribbon itself has a larger B-factor value and its conformation moves away from the catalytic center in the absence of ligand binding (Supplementary Figures S3C, D) (Cui et al., 2011). To assess the stability of the electrostatic interaction between SA and E71, we calculated the number of H-bonds between the two. All three replicate trajectories of the Holo system showed the presence of stable H-bonds between SA and E71, and the occupancy of H-bond_OE2-H14_ and H-bond_OE2-H13_ reached 86% (Figures 5D–F). To characterize the H-bonds, we calculated the electrostatic surface potentials of SA with E71. As shown in Figure 5G, there is an overlap of van der Waals surfaces in the regions where residues form H-bonds with SA, and the electrical properties of the overlapping regions are complementary (Figure 5G). As the H-bond between E71 and H40 is disturbed by SA binding, the H40 side chain no longer points toward residue C147 and is deflected away from the catalytic center by π-π stacking with SA (Figure 5H). The dihedral angle of H40 is deflected by about 100° (Figures 5I, J). The binding of SA leads to the allosteric of the 3C^pro^ catalytic triad, which ultimately prevents the 3C^pro^ from initiating the catalytic process.

### Binding and dissociation mechanism of SA and 3C^pro^

**FIGURE 6:**
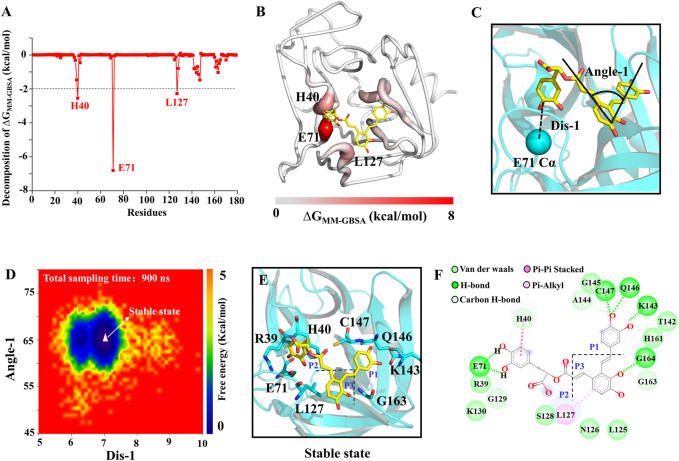
SA and 3C^pro^ binding mechanism. **(A)** The binding free energy decomposition of SA and 3C^pro^. **(B)** The binding free energy value mapping diagram (Where red indicates the regions contributing to ligand binding). **(C)** Schematic diagram of ligand reaction coordinates. **(D)** Free energy landscape of the SA binding mode. **(E)** and **(F)** representations of the binding mode of the SA and 3C^pro^ in their lowest binding energy conformation.

To assess the quantitative effect of affinities between the 3C^pro^ and SA, binding free energy calculation and decomposition were performed using the molecular mechanics generalized born surface area (MMGBSA) method. ΔG_MMGBSA_ of SA bound to the 3C^pro^ was −31.43 kcal/mol (Supplementary Table S3), and the ΔG_MMGBSA_ was driven by the electrostatic interaction (ΔE_ele_), polar solvation (ΔG_GB_), the vdW interaction (ΔE_vdW_), non-polar solvation (ΔG_SA_). Specifically, the contribution of ΔEele to the binding energy is the largest in this system (Supplementary Table S3). As in Figure 6A, the residues with the most favorable contributions (lower than −2.0 kcal/mol) to the binding free energy were labeled. According to the binding free energy decomposition spectrum, three residues that significantly promoted the binding were, E71 (−6.80 kcal/mol), H40 (−2.55 kcal/mol), and L127 (−2.28 kcal/mol). To determine the binding mode of SA to 3C^pro^, we mapped the free energy landscape of the SA conformation (Figure 6B). First, we calculated the distance between the E71 side chain and the SA terminal carbon atom, as well as the angle of the SA molecular structure (Figure 6C). We used these data as reaction coordinates to determine the lowest energy conformation of SA. The interaction mode showed a total of five types of interactions between SA and the 18 residues in the binding pocket, including van der Waals, H-bond interactions, carbon H-bonds, Pi-Pi stacked, and Pi-alkyl interactions (Figures 6D–F).

**FIGURE 7:**
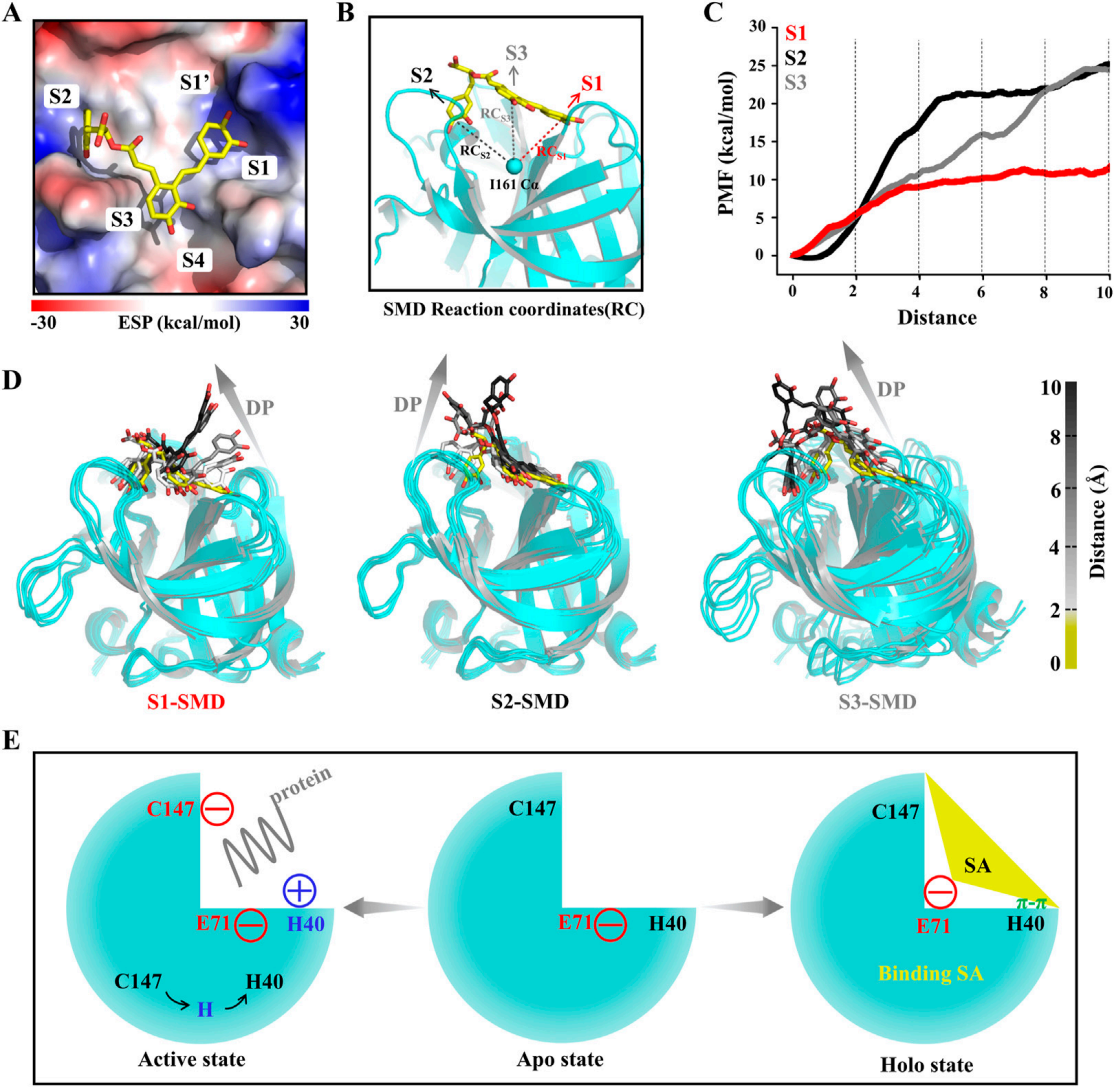
SA and 3C^pro^ dissociation mechanism. **(A)** The initial state conformation of steered molecular dynamics (SMD). **(B)** Schematic diagram of SMD reaction coordinates. **(C)** PMF profiles along the reactions coordinate for SA (*n* = 30). **(D)** Representative structures in the dissociation process of the SA from three pockets during SMD simulations. Dissociation path (DP). **(E)** Illustrations of the molecular mechanism of SA binding to 3C^pro^ and inhibition of its activity.

To explore the 3C^pro^-SA interactions and the affinity of the binding pockets for SA during SA dissociation, we performed steered molecular dynamics (SMD) simulations. Unlike the pore and groove binding models, the 3C^pro^ inhibitor binding pocket is located on the protein surface, and thus the SA molecule has a large degree of freedom and the dissociation pathway is difficult to determine. However, with limited calculations, each SA group can be made to dissociate from their respective pockets separately to determine the affinity of each group to the protein, which is critical for the druggability optimization of SA. Here, the PMF profile displayed the energy changes of SA each group unbinding to 3C^pro^ (Figures 7A–C). In the S1 pocket, the lower energy barrier (∼10 kcal/mol) makes it easier for the P1 group of SA to unbind (Figures 7C, D). The P2 group of SA requires the highest energy (∼25 kcal/mol) to unbind from the S2 pocket, indicating that this P2 has the highest affinity for the 3C^pro^, which is consistent with the estimate of the binding free energy (Figure 7D). It should be noted that the energy of the P3 group is not converged in 30 simulations, because the dynamic simulations show that the dissociation of the P3 group drives the P1 group to unbind from the S1 pocket together, which is determined by the structural rigidity of SA itself (Figure 7D).

## Discussion

HFMD has become a serious public health problem in the Asia-Pacific Region. Since May 2008, more than 13 million cases of HFMD have been reported cumulatively, including more than 3,300 deaths (Yang et al., 2017). Although the EV71 vaccine has been approved by the NMPA, there is an urgent need for efficient and safe antiviral drugs in the face of the mutation of the virus and the risk of its spread (Diarimalala et al., 2020). However, the drug progress has not been as developed as for vaccines, and still no relevant and effective drugs have been brought to market (Diarimalala et al., 2020). Currently, extensive research has focused on the structure of viral proteases and their interaction with synthetic inhibitors with a view to designing drugs that can combat the devastating epidemic of HFMD (Kuo et al., 2008). Considering the high safety and structural diversity of traditional Chinese medicine monomers, we screened an excellent antiviral small molecule, SA, using virtual screening as well as biological experiments targeting the 3C^pro^ of EV71. The results of molecular dynamics simulations show that SA can bind to a member of the catalytic triad, E71, and thus anchor to the protease substrate active site. And the binding of SA disrupts the interaction between E71 and H40, which in turn causes H40 to move away from the catalytic center, making the 3C^pro^ unable to initiate catalytic function (Figure 7E).

Indeed, disruption of the 3C^pro^ catalytic triad conformation is the key for the inhibitor to gain antiviral activity. The deflection of the H40 side chain was found as early as 2011 when using X-ray to resolve the complex of rupintrivir and 3C^pro^ (Wang et al., 2011). This is similar to the results found in our simulations, where a π-π stacking interaction was formed between H40 and the inhibitor P2 group. Differently from the inhibition mechanism of NK-1.8k, the P2 group of SA plays a key role in binding 3C^pro^, while the former anchors the sulfhydryl group of cysteine protease through the P1′ group aldehyde group (Wang et al., 2017a). The results of SMD simulations and MMGBSA calculations indicate that the P2 group can stabilize the binding conformation of the whole molecule by anchoring in the S2 pocket. Meanwhile, the binding and dissociation of ligands also point out that the P1 group of SA is the weakest bound to 3C^pro^, and it is the easiest to unbind to 3C^pro^. Based on the experience when optimizing NK-1.8K and NK-1.9K, the P1 group is very important for ligand activity, and replacing the P1 position of rupintrivir γ-lactam with δ-lactam not only increases the binding of the inhibitor to the target protein but also produces a higher hydrophobicity, which allows the compound to pass through the plasma membrane more easily (Wang et al., 2015; Wang et al., 2017a). In the future, if optimization and modification of SA molecules are needed to enhance the affinity of SA with 3C^pro^, it is recommended to enhance the binding energy of the P1 group to the S1 pocket.

Although our group has previously identified numerous small molecules that have inhibitory effects on EV71 infection, such as FOPMC/FIOMC (Xu et al., 2021), and SLQ-4/SLQ-5 (Liu et al., 2021), the pharmacology and toxicology of these compounds are unknown and these compounds require long-term testing and optimized modifications before they can hope to pass the evaluation phase. Here, we screened a compound with significant inhibitory activity against EV71 3C^pro^ based on a library of traditional Chinese medicine monomer compounds, SA. And, SA with its structural analogue (−)-Epigallocatechin gallate (EGCG) was reported to have significant inhibitory effect on SARS-CoV-2 3C^pro^ (Supplementary Figure S4) (Zhong et al., 2022). Also, the predicted targets of SA using SwissTargetPrediction showed that the protease was the predominant target of SA action (Supplementary Figure S5), and these results further support the molecular mechanism of SA as a viral protease inhibitor. SA is one of the major water-soluble phenolic acids extracted from *Salvia miltiorrhiza* (Li et al., 2008). *S.miltiorrhiza* has been used clinically to treat and prevent cardiovascular disease, hyperlipidemia, and cerebrovascular disease (Jiang et al., 2005). Notably, SA is one of the most potent compounds in *S. miltiorrhiza* that has the strongest protective effect against peroxidative damage to biological membranes. Studies have shown that SA has a variety of pharmacological activities, including prevention of brain lesions, defense from oxidative damage, and antithrombotic (Fan et al., 2010). Although the content of SA in *S. miltiorrhiza* is relatively low, some studies have shown that SA is more abundant in the Chinese drug agents, DanShenDiWan and FuFangDanShenPian (Sun et al., 2016). Moreover, Danhong injections containing SA have already entered clinical use, which indicates that the safety of SA in humans can be fully guaranteed. In conclusion, the discovery of SA will facilitate the research process of HFMD drugs and provide opportunities for the discovery of novel antiviral drugs.

## Experimental procedures

### Drug screening

The protein in the complex structure of EV71 3C^pro^ with NK-1.8K (PDB ID: 5GSO) (Wang et al., 2017a) was used as a docked receptor. Virtual screening of drugs was performed using the molecular docking program AutoDock Vina 1.0 (Trott and Olson, 2010). The traditional Chinese medicine database contains ∼2,300 monomers. Monomers with molecular weight >700 Da and <300 Da were removed. Autodock tool 1.5.6 (Morris et al., 2009) was used to prepare the PDBQT files of TMEM16A and drugs. The receptor was programmed to remain rigid, while the ligand was flexible. The grid center is determined according to the center of the binding pocket, with a searching space size of 24 × 24 × 20 Å^3^. The global search exhaustiveness value was set to 50. The maximum energy difference between the optimal binding mode and the worst case was set to 5 kcal/mol to ensure diverse docked poses. The test molecules were purchased from MedChemExpress (MCE) and the purity of SA was 99.75%. SwissTargetPrediction (Daina et al., 2019) was used to predict the possible protein targets of the molecules.

### Molecular dynamics simulations and quantum chemical calculations

The simulation systems were constructed using tleap program of Amber 16 (Case et al., 2016). The simulation boxes contained approximately 33,000 atoms and dimensions of ∼76 × 73 × 74 Å^3^. All simulations were performed using Amber16 (Case et al., 2016). The Amber ff14SB force field and the Joung/Cheatham ion parameters (Li et al., 2013; Li and Merz, 2014) were used. Parametrization of SA was performed using the Antechamber module of Amber16, using the Generalized Amber Force Field to assign atom types and the AM1-BCC method to assign charges. First, the simulated system of solutions, and the entire system were sequentially performed for energy minimization. Next, the system temperature was increased from 0 to 100 K under the NVT ensemble, and then the temperature was increased from 100 to 300 K under the NPT ensemble, during which the protein was restraint (1 kcal mol^−1^·Å^−2^). Finally, for each simulation system, three separate production simulations were performed under NPT conditions at 300 K and 1 bar. The other parameters were the same as we set before (Shi et al., 2021; Shi et al., 2022). The snapshots were extracted every 100 ps for all equilibrium MD trajectories to calculate statistical distributions. The CPPTRAJ module of the Amber 16 program was used to analyze the generated trajectories. Quantum chemical calculations were performed using Gaussian 03 (M. J. Frisch et al., 2003) and Multiwfn (Tian and Feiwu, 2012) programs. The wave function data used in the electrostatic surface potential analysis were generated using the B3LYP/6–31G** level algorithm.

MMPBSA.py in the AmberTools16 package (Miller et al., 2012) was employed to conduct free energy calculations for the two complexes. 100 conformations were extracted from each equilibrious trajectory (from 250 to 300 ns) for calculations. SMD simulations (Jensen et al., 2002) were performed using the Amber16 software package. Here, SA with 3C^pro^ stable state was selected as the initial conformation of SMD. To obtain the converge potential mean of force (PMF) in SMD, SA was simulated 30 times along each of the three reaction directions. In these simulations, the trajectories with energy values closest to the Jarzynski average (JA) are considered representative. The stretching velocity was 10 Å/ns in this SMD simulation, coupling a spring constant k of 40 kcal/(mol × Å^2^). As the distance between the two selected atoms reached 10 Å, there is no longer any interaction between the ligand-related groups and the corresponding binding pocket.

### Preliminary screening of antiviral activity

The inhibitory activity against EV71 of traditional Chinese medicine monomers was evaluated by phenotype screening. Briefly, 3 × 10^4^ RD cells were seeded in a 96-well plate and cultured overnight at 37°C in 5% CO_2_. Monomers (10 µM) and EV71 luciferase virus (MOI = 1) were added and incubated for 24 h. The luciferase expression level was monitored using a Microplate Reader (Tecan, Austria).

### *In vitro* inhibition assay

The fluorescent peptide NMA-IEALFQGPPK(DNP)FR was employed as the substrate for inhibition assay based on the FRET effect. The inhibition assay proceeded to contain 1 μM EV71 3C^pro^, 20 μM substrate and 1 μM SA in 50 mM HEPES (pH 7.5), 100 mM NaCl, 2 mM DTT at 30°C. The fluorescence intensities were read at λex = 340 nm and λem = 440 nm every 1 min for 60 min. The IC_50_ was executed with gradient diluted SA (0.2–50 μM) and incubated at 30°C for 2 h, and then 20 μM substrates were added into each well. The fluorescence intensities were read at a Microplate Reader and calculated by GraphPad Prism 7.0.

### The inhibition effect and cytotoxicity of SA

RD (3 × 10^4^ per well), Vero (3 × 10^4^ per well), and HEK-293T (2 × 10^4^ per well) cells were seeded in 96-well plates and cultured at 37°C 5% CO_2_ overnight. Each cell line was treated with serial dilutions of the SA ranging from 0.05 to 50 μM. EV71 luciferase reporter virus was added after 2 h and cultured for 24 h. The supernatants were removed, and cells were lysed using Bright-Glo Luciferase substrate. The luciferase values were read on a Microplate Reader (Tecan, Austria), and the EC_50_ was calculated using Graph Pad Prism. Cell viability assay was used to measure the cytotoxicity of SA on different cell lines. Serial dilutions of the SA (1.56–100 μM in DMEM) were added and incubated for 48 h at 37°C. Cells were incubated for 10 min with 100 μL of CellTiter-Glo^®^ reagent (Promega, United States). The luminescence signals were determined using the Microplate Reader. The viability of cells treated with inhibitors was relativized to that of the non-treated cells.

### Time of addition assay

We performed the time of addition assay with SA, NK-1.8k, and GPP3 to elucidate the stage at which the compound inhibited viral replication. RD (3 × 10^4^) cells were cultured in 96-well plates at 37°C under 5% CO_2_ overnight. Cells were treated with 5 μM SA, 2 µM NK-1.8k, and 1 µM GPP3, respectively, and infected with EV71 luciferase reporter virus for different periods. After 24 h post-infection (hpi), antiviral activity was determined by the reduction of the luciferase activity compared with the control cultures using Bright-Glo Luciferase substrate.

### Data analysis

Graphical presentation and data analysis were performed using Microsoft Excel 2019. The data are presented as mean ± standard deviation (S.D.), and the number of replicates is given in Figure legends. Statistical significance of the differences between group means was evaluated by one-way analysis of variance (ANOVA) using Tukey’s honestly significant difference (HSD) test as a *post hoc* test; *p* values ≤0.05 were considered statistically significant (*p < 0.05, **p < 0.01). Discovery Studio visualizer was used to analyze non-covalent interactions between SA and its binding pocket. Visualization and analysis of model features were performed by VMD (Humphrey et al., 1996) and Open-Source Pymol (https://pymol.org).

## Data Availability

The original contributions presented in the study are included in the article/Supplementary Material, further inquiries can be directed to the corresponding authors.

## Abbreviations

3C^pro^, 3C Protease; EC_50_, the half maximal effective concentration; ESP, electrostatic surface potential; EV71, Enterovirus 71; HEK-293T, Human embryonic kidney cells; HFMD, Hand, foot, and mouth disease; IC_50_, the half maximal inhibitory concentration; MD, Molecular dynamics; MMGBSA, molecular mechanics generalized born surface area; RD, Human malignant embryonic rhabdomyoma cells; RMSD, root mean square deviation; SA, Salvianolic acid A; SMD, steered molecular dynamics; Vero, African green monkey kidney cells.
