# Supplementary material for: Computational and experimental studies of salvianolic acid A targets 3C protease to inhibit enterovirus 71 infection
Source: Front Pharmacol. 2023 Mar 2;14:1118584. doi: 10.3389/fphar.2023.1118584 (PMC10017496; doi:10.3389/fphar.2023.1118584)
Supplement: Supplementary file 1 [file DataSheet1.PDF]

Supplementary Information

Computational and Experimental Studies of Salvianolic acid A Targets 3C Protease to Inhibit Enterovirus 71 Infection

Sai Shi 1†, Lei Xie 1†, Sen Ma 1, Binghong Xu 1, Hailong An 2, Sheng Ye 1*, Yaxin Wang 1*

1 Tianjin Key Laboratory of Function and Application of Biological Macromolecular Structures, School of Life Sciences, Tianjin University, Tianjin, 300072, China

2 Key Laboratory of Molecular Biophysics of Hebei Province, Institute of Biophysics, Hebei University of Technology, Tianjin, 300401, China

* Corresponding authors:

Sheng Ye, School of Life sciences, Tianjin University, Tianjin 300072, China, Email: sye@tju.edu.cn;

Yaxin Wang, School of Life sciences, Tianjin University, Tianjin 300072, China, Email: wangyaxin@tju.edu.cn.

† These authors contributed equally to this work.

Running title: Salvianolic acid A targets $3C^{pro}$ to inhibit EV71

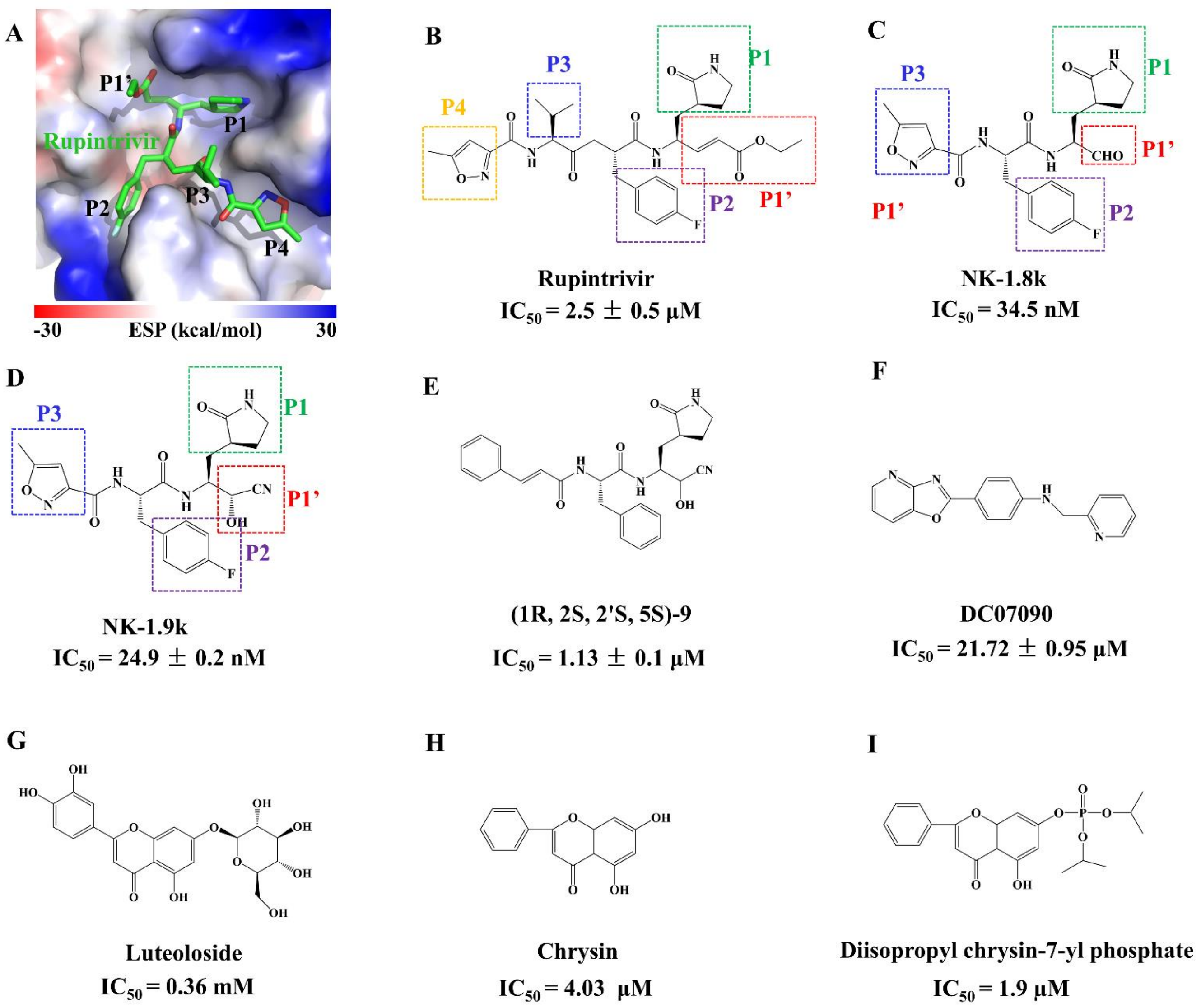

Fig S1. The structure and IC_{50} values of EV71 $3C^{pro}$ inhibitors. (A) Binding mode of Rupintrivir and EV71 $3C^{pro}$. **(B-I)** The structures of some 3C inhibitors have been reported as well as the IC_{50} for inhibiting $3C^{pro}$.

Table S1. Affinity assessment of candidates

Candidates	binding energy (kcal/mol)
Amentoflavone	-9.1
Didymin	-9
Linarin	-8.8
Narirutin	-8.7
Rhoifolin	-8.7
Luteolin-7-glucuronide	-8.7
Procyanidin B2	-8.7
Methyl hesperidin	-8.6
Salvianolic acid A	-8.6
Scutellarin	-8.6

Table S2. Configuration of the simulation system

System Name	Number of samples	Time
Apo system	300 ns * 3	900 ns
Holo system (H40: HID)	100 ns * 3	300 ns
Holo system (H40: HIE)	300 ns * 3	900 ns
SMD	1000 ps* 30*3	30000 ps* 3

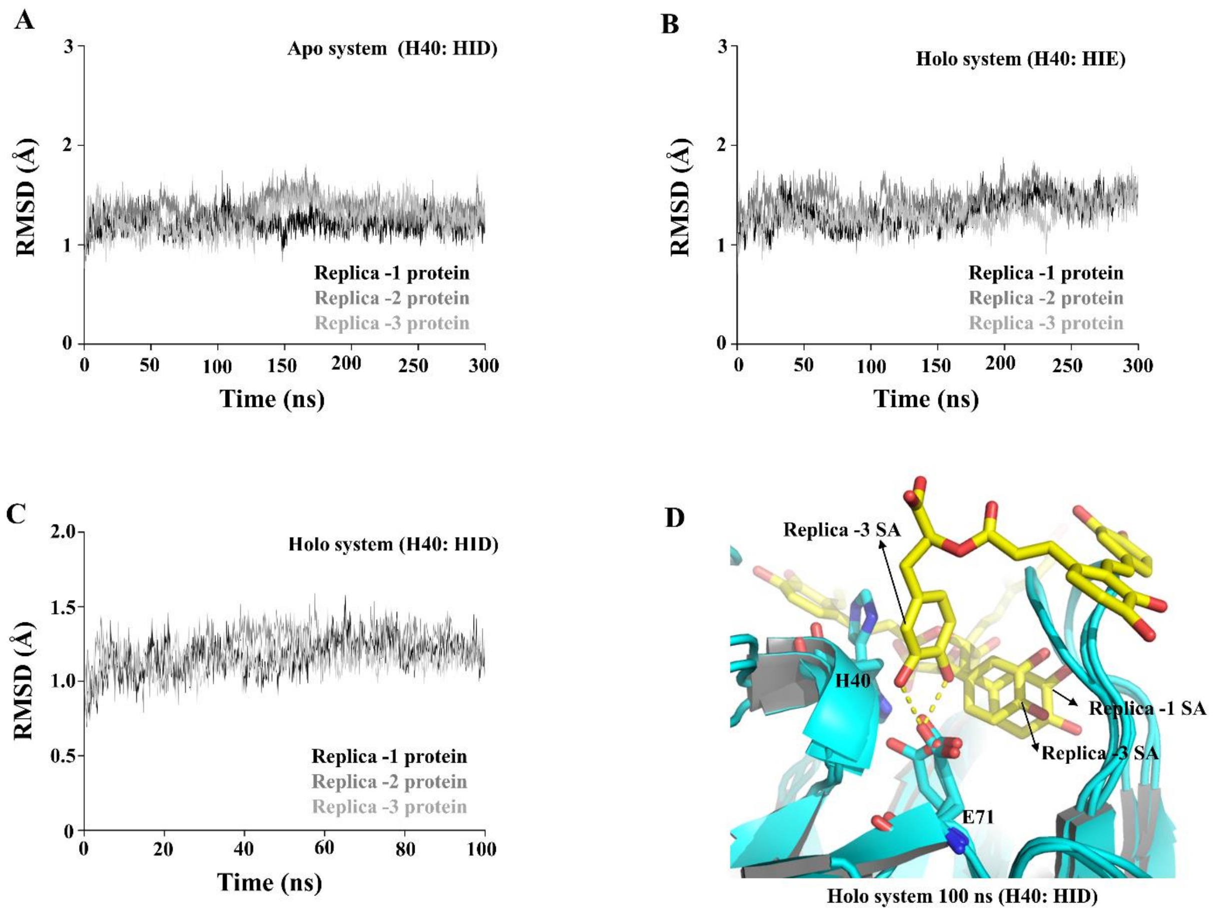

Fig S2. RMSD for each simulation system. **(A)** **(B)** and **(C)** RMSD for each simulation system. **(D)** Overlap of the structures of the 3C^{pro} in different states. **(D)** H-bond between SA and E71 at 100ns in Holo system (H40: HID).

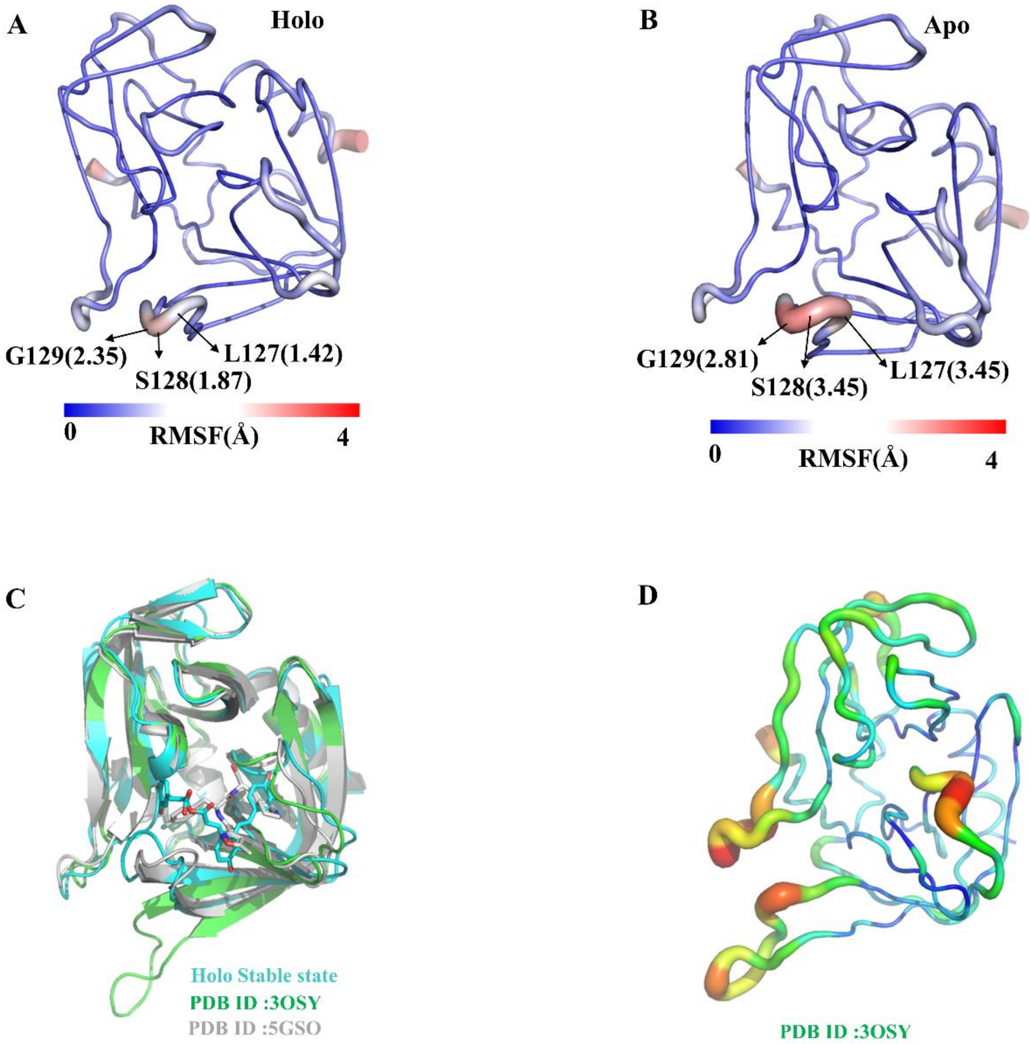

Fig S3. Flexibility of 3C^{pro} structure. **(A)** and **(B)** The RMSF value mapping diagram (where red indicates flexible regions, and blue indicates rigid regions). **(C)** Overlap of the structures of the 3C^{pro} in different states. **(D)** The B-factor of 3OSY (Apo state).

Table S3. MMGBSA binding free energy calculation

Energy Component	Average	Std. Err. of Mean
ΔE_{vdw}	-33.0226	4.083221336
Δ_{Eelc}	-52.51593333	1.772876266
ΔE_{polar}	59.307	0.421253024
$\Delta E_{nopolar}$	-5.2011	0.364482853
ΔG_{MMGBSA}	-31.43256667	4.783588649

ΔG_{MMGBSA}=ΔE_{vdw} +Δ_{Eele}+ΔE_{polar} + $\Delta E_{nopolar}$

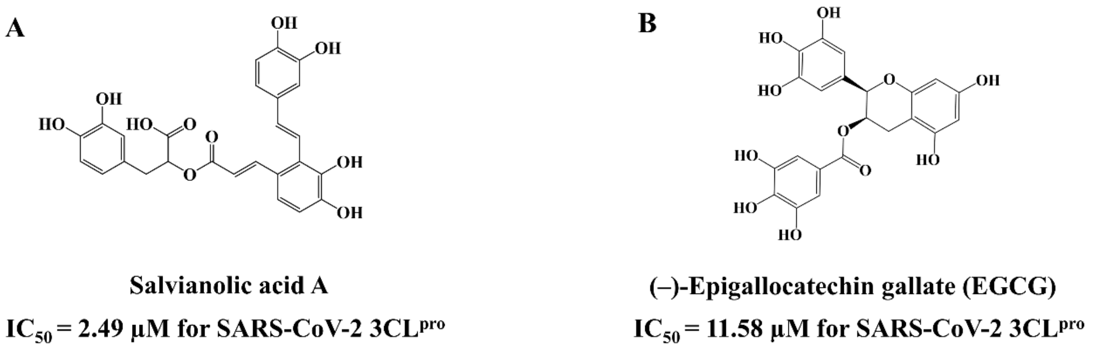

Fig S4. Structure of salvianolic acid A / (−)-Epigallocatechin gallate and its IC_{50} for inhibiting SARS-CoV-2 $3C^{pro}$. (A) salvianolic acid A. **(B)** (−)-Epigallocatechin gallate (EGCG).

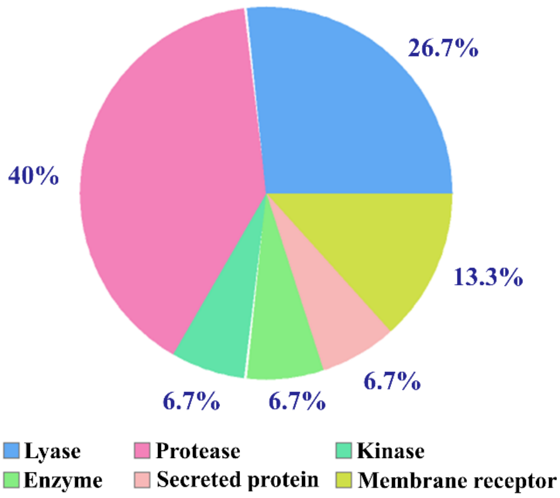

Fig S5. The probable protein targets of SA predicted by SwissTargetPrediction. Proteases and lyase represent the most likely targets of SA.
